# A model for the impact of FFPE section thickness on gene copy number measurement by FISH

**DOI:** 10.1038/s41598-019-44015-7

**Published:** 2019-05-17

**Authors:** Jiyan Yu, Qi Wang, Pu Xue, Li Zheng, Juanfen Mo, Liangye Chen, Manxiang Yin, Yueyan Huang, Yi Bao, Feng Ding

**Affiliations:** 1 Department of Biotechnology and Biomedicine, and Zhejiang Provincial Key Laboratory of Applied Enzymology, Jiaxing ACCB Diagnostics, Yangze Delta Regional Institute of Tsinghua University, Zhejiang, Jiaxing, 314006 China; 2 Department of Research Center, The Second Hospital of Jiaxing, Jiaxing, China; 3 Department of Pathology, Zhejiang Provincial Corps Hospital of Chinese People’s Armed Police Force, and Medical College of Jiaxing University, Jiaxing, 314000 China

**Keywords:** Cancer genetics, Tumour biomarkers

## Abstract

Fluorescent *in situ* hybridization (FISH) assays to detect gene amplification such as HER2 or MET in tumors are used for prognosis evaluation and selection of targeted therapies. Although FISH guidelines recommended 4~6 μm FFPE sections, many laboratories use 2~3 μm sections, which is a common practice for H&E staining and immunohistochemistry. A former study concluded that section thickness did not affect FISH results. We found, however, that thinner FFPE sections may lead to false negative results for gene amplification. A mathematic model was constructed and cell-line based controls with known gene copy number were prepared, and the model had a reasonable fit with the experimental data. The model revealed that even when counting the apparently full-sized nuclear images, many of them have partial volumes, which leads to under-estimation of gene copy number. Therefore, improperly thinner sections are prone to give false negative results, and thicker sections give a better approximation to the true value. The discrepancy between this and the former study was discussed. In summary, the model applies generally to FISH/ISH detection of gene copy number, and section thickness is an important parameter to control for precision medicine research, assay development, clinical trials and daily practice in pathology laboratory.

## Introduction

Gene amplification is a common mechanism for oncogenic driver mutation, and the amplifications of genes such as HER2/ERBB2, MET, EGFR and MYC are associated with prognosis, dosage optimization for chemotherapy, and selection for targeted therapies. For instance, trastuzumab therapy for metastatic breast carcinoma and gastric carcinoma requires HER2-postive status, with evidence of either protein overexpression by immunohistochemistry (IHC) or gene amplification by fluorescent *in situ* hybridization (FISH)^1–4^. In non-small-cell lung carcinoma (NSCLC), patients with MET amplification as assessed by FISH^5–7^ had been shown to contribute to resistance to gefitinib therapy and worse prognosis, but benefit from crizotinib^6–8^. In addition, MET amplification is also implicated as a poor prognosis factor for gastroesophageal adenocarcinoma^9^ and ovarian cancer^10^. EGFR amplification has recently been shown to correlate with the outcome of afatinib treatment in NSCLC^11^, as well as for the combination therapy with cetuximab in squamous NSCLC patients^12^.

To ensure accurate measurement of gene amplification, professional organizations and clinical communities developed guidelines and recommendations for FISH/ISH assays, such as those for HER2 in invasive breast carcinoma^2–4^ or gastroesophageal adenocarcinoma^1^, and MET in non-small-cell lung carcinoma^5^. Both the ratio of the oncogene to control probe and/or the copy number of the oncogenes were used as criteria for gene amplification status by FISH/ISH assays.

In the first companion diagnostic FISH test for HER2 amplification in breast cancer, the ratio of HER2 to chromosomal 17 centromere probe (CEP17) ≥ 2.2 is used as criteria for positive result. Later studies showed that chromosome 17 polysomy, which occurred in about 8%~12% cases of invasive breast cancer, may lead to HER2 overexpression^13–15^. In the 2007 ASCO/CAP guideline for HER2 assay in breast cancer, the criteria for amplification was expanded to include HER2 copy number ≥6 in tests without CEP17 probe^3^. The 2013 ASCO/CAP guideline^2^ is more inclusive, in which the HER2/CEP17 ratio ≥2.0 or the HER2 copy number ≥6 are defined as positive. Recently, the 2018 update combined FISH and IHC to resolve equivocal results^4^, in which the HER2 copy number ≥6 is still used as the criteria for gene amplification.

As for MET amplification, Cappuzzo et.al showed that the copy number of MET (≥5 copies) is an independent negative prognostic marker for NSCLC^7^. Recent studies showed that the absolute copy number of MET^16^ and chromosome 7^17^ are independent prognosis factors in NSCLC. Moreover, the absolute copy number of c-MET but not MET/CEP7 ratio was found to be an indicator for therapeutic response to crizotinib in lung adenocarcinoma^8^.

The criteria for EGFR amplification in tumors as measured by FISH/ISH uses both the ratio of EGFR to chromosome 7 and the absolute copy number of EGFR. Patients with either high polysomy of chromosome 7 or focal EGFR amplification of ≥4 copies were considered FISH positive^11,12^. Taken together, these studies showed that the correct measurement of absolute copy number for genes such as HER2, MET and EGFR are important for clinical studies and patient care.

In our practice, FISH is performed on specimens from different hospitals. We noticed that specimens from some hospitals were prone to over-digestion during pretreatment. Upon inquiry, it was found that 2 or 3 μm FFPE sections routinely used for H&E and IHC, which deviated from recommended 4 to 6 μm for FISH^2–4^, had been provided. Hence, the influence of section thickness on FISH assay was investigated, and a mathematic model was established. The model had a reasonable fit with the experimental data, and revealed that section thickness correlates with the number of partial nuclei on the slide, which subsequently influences gene copy number measurement.

## Results

### Section thickness influenced the FISH result of clinical specimens

**Figure 1:**
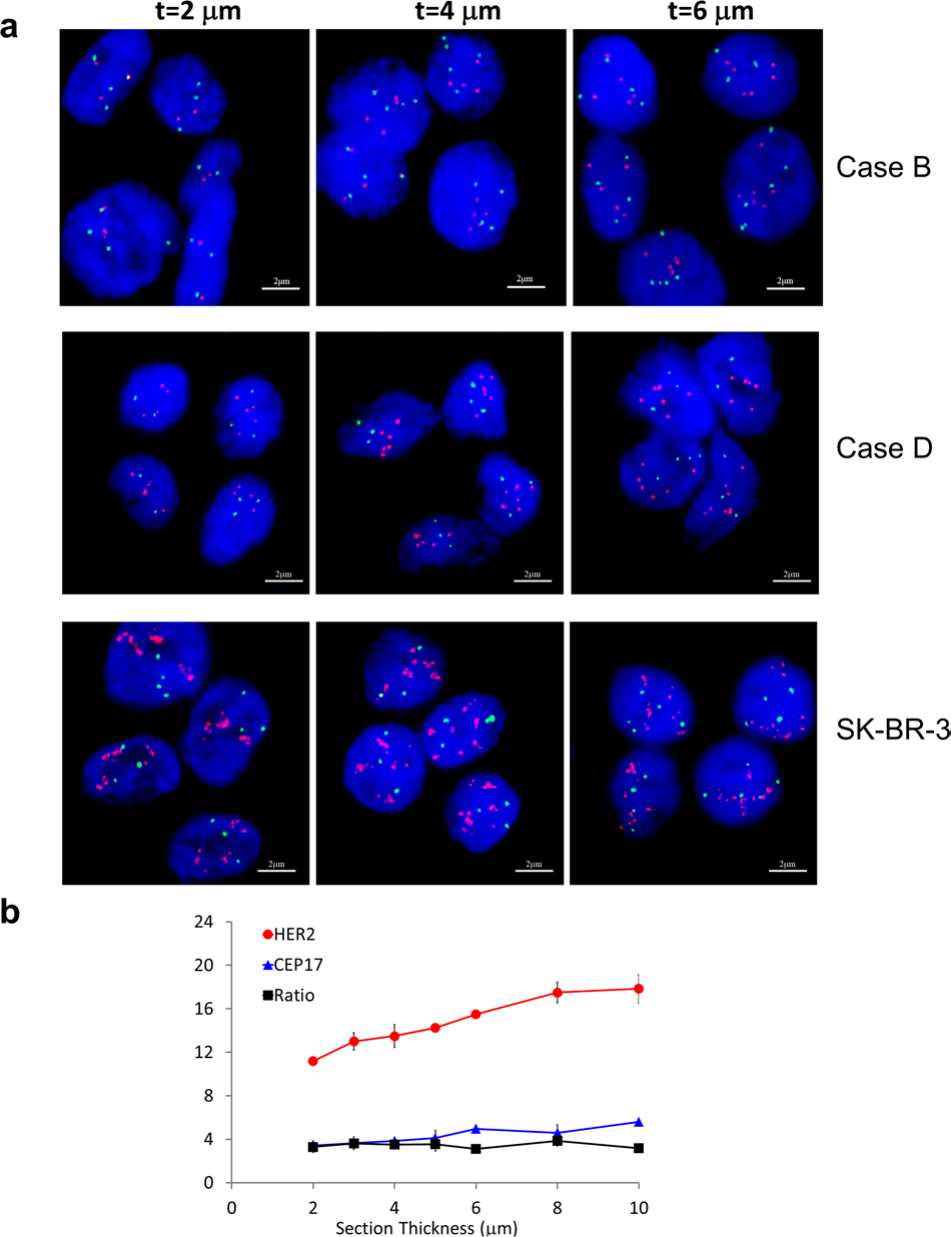
The influence of FFPE section thickness on fluorescence *in situ* hybridization result. (**a**) Human breast cancer specimen Case B and D, as well as FFPE sections of SK-BR-3 cells were section to different thicknesses, and FISH experiment was performed using HER2 (Orange red) and CEP17 (green) probes. The representative images for 2, 4, and 6 μm sections were shown. (**b**) Observed gene copy numbers for HER2, CEP17 and HER2/CEP17 ratio using 2 to 10 μm FFPE sections of SK-BR-3 cells. Red curve, observed HER2 copy number; blue curve, CEP17 copy number; black curve, HER2/CEP17 ratio. The error bars represent standard deviation from triplicate experiments.

Breast cancer specimens were sectioned to 2, 4 and 6 μm in triplicates. Using 4 μm sections as recommended by ASCO/CAP guidelines^2–4^, the HER2 status were negative (Case A), equivocal (Case B) and positive (Case C and D), respectively (Table 1). As shown in representative images for Case B and D (Fig. 1a), when the section thickness decreased to 2 μm, the HER2 copy number of Case B decreased from 4.4 to 3.4, and the apparent FISH result changed from equivocal to negative. And for Case D, the apparent FISH result changed from positive to equivocal.

**Table 1:** Observed HER2 and CEP17 copy numbers of human breast carcinoma specimens using FFPE sections of different thickness.

Specimen	HER2 status	HER2		CEP17		HER2/CEP17	
		Ave.	CV	Ave.	CV	Ave.	CV
*Case A*							
2 μm	negative	2.2	9.2%	2.0	19.5%	1.1	7.9%
4 μm	negative	2.4	8.8%	2.4	14.9%	1.1	8.2%
6 μm	negative	3.0	7.6%	2.8	10.2%	1.1	7.5%
*Case B*							
2 μm	**negative**	3.4	22.0%	2.4	13.3%	1.4	13.9%
4 μm	equivocal	4.4	20.2%	3.6	25.9%	1.2	5.2%
6 μm	equivocal	4.4	18.8%	3.6	15.5%	1.2	3.9%
*Case C*							
2 μm	positive	7.9	34.0%	2.3	37.2%	3.5	3.3%
4 μm	positive	11.4	19.7%	3.0	18.7%	3.9	2.9%
6 μm	positive	12.6	13.2%	3.8	12.3%	3.3	2.8%
*Case D*							
2 μm	**equivocal**	4.8	22.2%	2.55	19.0%	1.88	6.3%
4 μm	positive	5.8	17.1%	2.65	17.9%	2.19	7.4%
6 μm	positive	6.4	18.8%	2.84	16.6%	2.25	6.2%

Average copy numbers (Ave) and coefficient of variation (CV) of HER2, CEP17 and HER2/CEP17 ratio were obtained from triplicate slides. The ANOVA tests for gene copy number of different section thickness did not reach statistical significance.

Serial sections were also prepared for negative and positive cell-line control specimens as shown in Table 2, with representative images of Positive control 2 (FFPE sections of SK-BR-3 cell line) shown in Fig. 1a and curves in Fig. 1b. With decreasing thickness, the observed copy numbers for HER2 and CEP17 were significantly decreased for both Positive control 1 and 2, whereas the HER2/CEP17 ratios were stable.

**Table 2:** Observed HER2 and CEP17 copy numbers of negative and positive control FFPE specimens made from cell lines using sections of different thickness.

Specimen	HER2 status	HER2		CEP17		HER2/CEP17	
		Ave.	CV	Ave.	CV	Ave.	CV
*Negative control (293T cell line)*							
2 μm	negative	2.2	8.7%	2.2	18.4%	1.0	12.9%
3 μm	negative	2.1	10.7%	2.1	3.6%	1.0	9.8%
4 μm	negative	2.4	19.6%	2.4	26.4%	1.0	9.6%
5 μm	negative	2.5	5.9%	2.3	9.8%	1.1	4.2%
6 μm	negative	2.5	16.5%	2.4	25.0%	1.1	9.7%
*Positive control 1 (1:1 mix of 293T and SK-BR-3 cell lines)*							
2 μm	positive	8.0	13.0%	3.0	3.8%	2.6	9.9%
3 μm	positive	8.2	19.6%	2.6	3.7%	3.1	16.4%
4 μm	positive	10.1	15.8%	3.0	3.7%	3.4	12.3%
5 μm	positive	11.7	10.3%	3.3	13.1%	3.5	12.9%
6 μm	positive	12.3	4.1%	3.8	7.8%	3.3	9.9%
*Positive control 2 (SK-BR-3 cell line)*							
2 μm	positive	11.2	3.5%	3.4	11.9%	3.3	14.8%
3 μm	positive	13.0	6.1%	3.6	15.7%	3.6	9.1%
4 μm	positive	13.5	7.8%	3.9	5.8%	3.5	6.7%
5 μm	positive	14.3	1.9%	4.1	17.0%	3.5	17.7%
6 μm	positive	15.5	2.2%	5.0	6.4%	3.1	4.3%
8 μm	positive	17.5	5.5%	4.6	15.7%	3.8	11.8%
10 μm	positive	17.8	7.4%	5.6	4.8%	3.2	7.9%

Average copy numbers (Ave) and coefficient of variation (CV) of HER2, CEP17 and HER2/CEP17 ratio were obtained from triplicate slides. The ANOVA test for Positive control 1 series showed significant difference in gene copy numbers (*p* = 0.006 for HER2, *p* = 0.002 for CEP17) but not HER2/CEP17 ratio among sections of different thickness. Positive control 2 series also showed significant difference in gene copy numbers (*p* = 0.0000008 for HER2, *p* = 0.001 for CEP17) but not HER2/CEP17 ratio.

Moreover, a non-small cell lung cancer specimen was examined by using MET/CEP7 probes (Table 3). Though the FISH results at different thickness were all negative, significant lower copy number was observed for MET on sections of different thickness.

**Table 3:** Observed MET and CEP7 copy numbers of a human lung cancer specimen using FFPE sections of different thickness.

Specimen	MET status	MET		CEP7		MET/CEP7	
		Mean	CV%	Mean	CV%	Mean	CV%
2 μm	negative	2.4	15.5	2.0	8.1	1.2	7.6
4 μm	negative	3.1	13.1	2.6	14.3	1.2	1.5
6 μm	negative	3.5	8.3	2.9	16.1	1.2	8.4

Average copy numbers (Ave) and coefficient of variation (CV) of MET, CEP7 and MET/CEP7 ratio were obtained from triplicate slides. The ANOVA test showed significant difference in gene copy numbers for MET (*p* = 0.023) but did not reach significance for CEP7 (*p* = 0.060) among sections with different thickness.

In summary, for the above cases, a trend of lower gene copy number is observed with decreasing section thickness.

### Mathematic model for the relationship of FFPE section thickness and observed gene copy number

**Figure 2:**
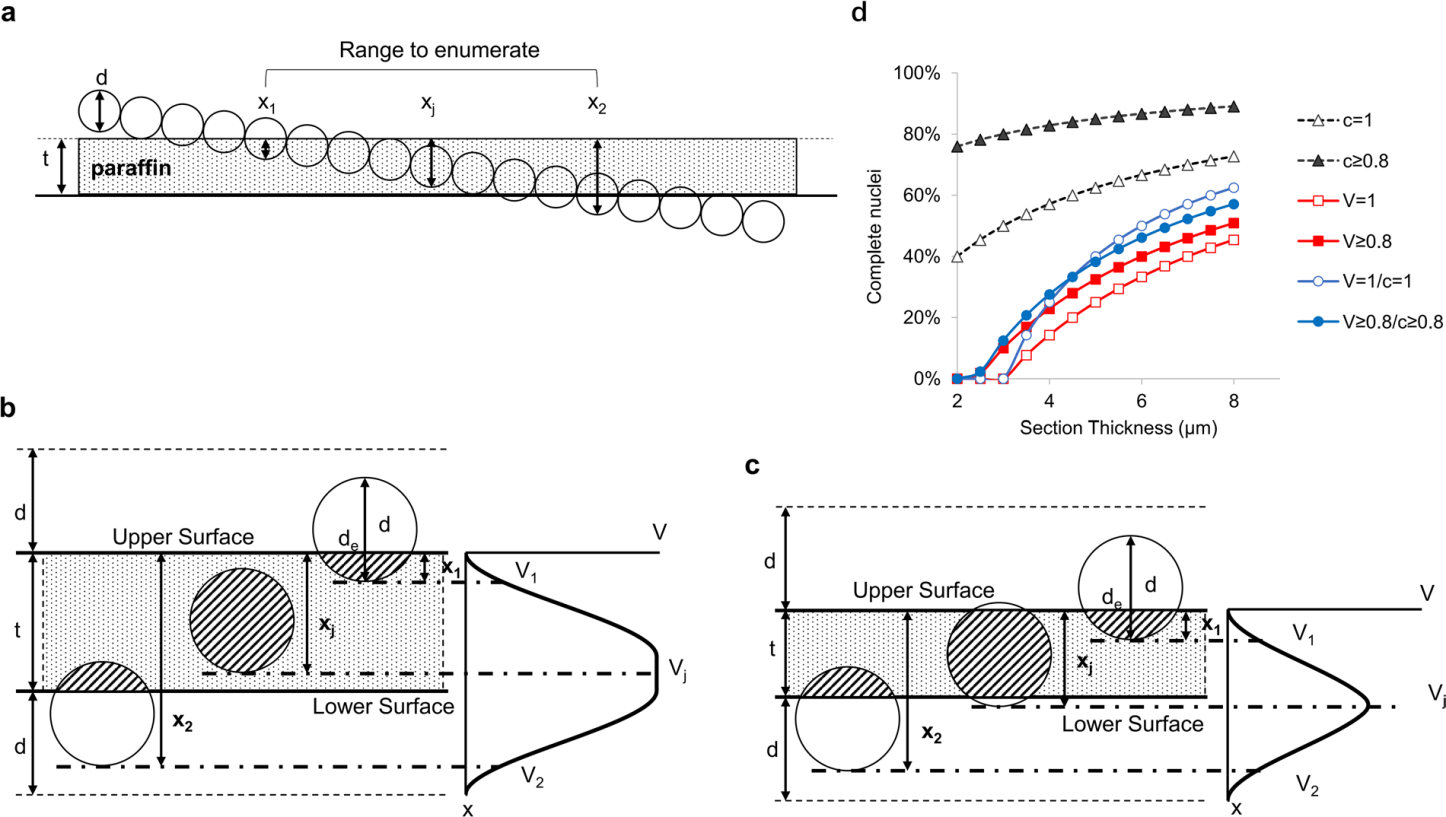
Model for FISH signal distribution in paraffin sections of different thickness. (**a**) Schematics of the distribution of nuclei in paraffin section. The nuclear diameter is *d*, and section thickness of paraffin is *t*. The distance between the upper surface of the section and the lowest point of a nucleus is defined as depth *x*. (**b**) Scenario when section thickness is greater or equal to nuclear diameter (*t* ≥ *d*). The volume of the partial nucleus in the paraffin block is *Vx*. The acceptable nuclear diameter is *d*_*e*_, and the distribution of the volume of the nuclei retained on the slide is shown as the curve on the right. Nuclei with volume from *V1* to *V2* were counted. (**c**) Scenario when section thickness is less than the nuclear diameter (*t* < *d*). (**d**) Percentage of complete nuclei on sections of different thicknesses based on the model. The black curves represent percentage of nuclei with expected diameter in microscopic images (*c* = 1, nuclei with full diameter, in this example *d* = 3 μm was used; *c* ≥ 0.8, nuclei with ≥80% of the full diameter). The red curves represent percentage of nuclei with expected volume (*V* = 1, nuclei with full volume; *V* ≥ 0.8, nuclei with ≥80% of the full volume). The blue curves represent percentage of nuclei with expected volume among those with certain diameters (*V* = 1/*c* = 1, percentage of nuclei with full volume among those with full diameter; *V* ≥ 0.8/*c* ≥ 0.8, percentage of nuclei with ≥80% full volume among those with ≥80% full diameter).

To understand the impact of section thickness on FISH assay for gene amplification, a mathematic model was constructed. For simplicity, nuclei were regarded as spheres and the gene signals were assumed to have a random distribution in the nucleus. As shown in Fig. 2a, when sectioning a paraffin block, many partial nuclei are retained on the slide, and the observed gene copy number for each nucleus is proportional to the residual nuclear volume.

The calculation of average nuclear volume on a slide is illustrated in Fig. 2b,c, and is described in Eqs (1–4) (see Material and Methods). If the actual gene copy number is *N*, the probability of observing a certain copy number *n* is defined by Eqs (8–10), with *N*_*G*_ and *n*_*G*_ being the actual and observed copy number of target gene, and *N*_*C*_ and *n*_*C*_ being those of the control, respectively. To mimic human observers, a nucleus is enumerated only when its image has a diameter above a certain fraction *(c)* of the full-sized ones.

In Fig. 2d, the black curves indicated the percentage of nuclear images that appeared complete (*c* = 1) or roughly complete (*c* ≥ 0.*8*, the area is greater than 64% of the full-sized image). Counterintuitively, even if only “complete” nuclear images are counted (*c* = *1)*, not all has a full volume. A nucleus sectioned at equator has a full-sized image but only half the volume. When *t* < *d*, none of the nuclei has a full volume, although many appear as full-sized images. On 2 to 8 μm sections, the percentage of full-sized images (*c* = *1*.*0*) ranged from 40% to 73%, and those close to full-sized images (*c* ≥ *0*.*8*) ranged from 76% to 89%. The portion of nuclei with a full volume (*V* = *1*.*0*) or close to full diameter (*V* ≥ *0*.*8*) among images with roughly complete diameter (*c* ≥ *0*.*8*) ranged from 0 to 51% and 0 to 57%, respectively. Hence, even if only the full-sized or roughly full-sized nuclear images were counted, which includes 100% to 43% partial nuclei, the observed results would still be an underestimation of the true gene copy number.

### Probability plots for sections of different thickness

To test the mathematic model, control sections were prepared from culture cells with and without HER2 amplification. The HER2 and CEP17 copy numbers in intact cells were measured using slides prepared from cell suspension by a cytology procedure. The average copy numbers of a positive cell line, SK-BR-3, were 22.9 ± 4.8 for HER2 and 6.5 ± 1.7 for CEP17, in line with the literature^18–20^. The average copy numbers of a negative cell line, HEK293T, were 3.04 ± 0.78 for HER2 and 3.09 ± 0.71 for CEP17. The FFPE block of positive control 1 were made from 1:1 mixture of HEK293T and SK-BR-3 cells. Hence, the theoretical values for HER2 and CEP17 of the positive control were 13.0 and 4.8, respectively.

Under fluorescence microscope, the average diameter of DAPI-stained nuclei after FISH experiment was 3.65 ± 0.45 μm for the positive control slides. To estimated how many nuclei were made partial during sectioning, the nuclear diameter (*d*) of the original FFPE block was measured by DAPI staining of the dewaxed and rehydrated slides. The nuclear size was 3.35 ± 0.41 μm, which is 8% smaller, suggesting that nuclear DNA might have been loosened upon protease treatment and/or hybridization during FISH assay.

**Figure 3:**
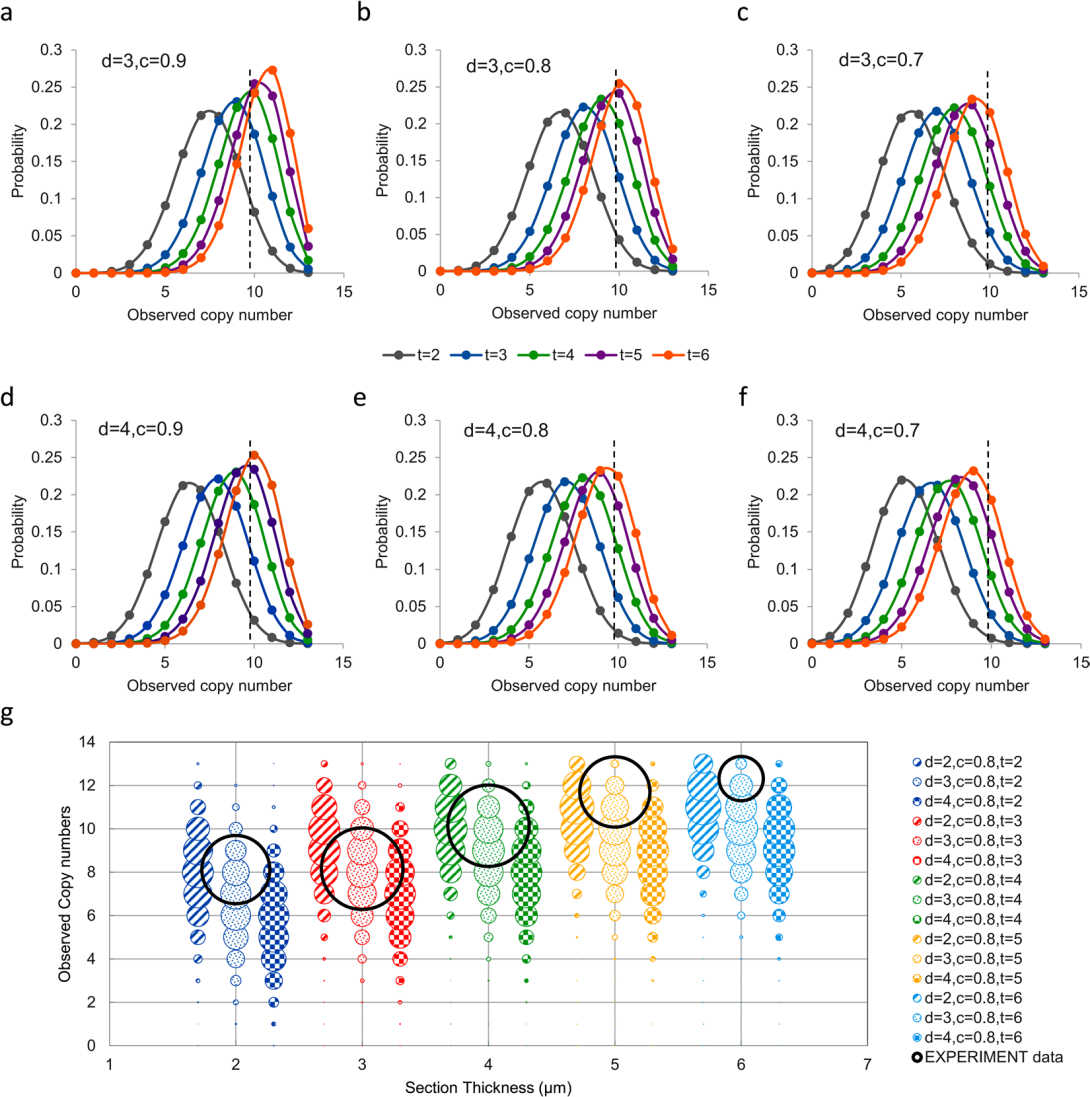
Probability plots of the observed gene copy number for samples with different nuclear diameter and section thickness. (**a**–**f**) Probability distribution of observed copy number for the positive control 1 that has a theoretical HER2 copy number of 13. With section thickness of 3 μm (**a**–**c**) or 4 μm (**d**–**f**), different enumeration thresholds (*c* = 0.7, 0.8, 0.9) were used. The vertical dashed line represents the observed copy number using 4 μm section. (**g**) Comparison of experimental data with model prediction at different section thickness. The colored bubbles with different patterns represent the probabilities of observed gene copy number as predicted by the mathematical model when theoretical copy number is 13. The bubble center represents a certain observed *n*_*G*,_ and the bubble size represents the probability of obtaining that *n*_*G*_. The experiment data were shown as black circles, with their diameters reflecting the standard deviation of triplicate experiments.

To mimic human observers, *c* values of 0.7–0.9 were used to construct the theoretical curves, which correspond to images with apparent area of 49~81% of the full-sized images. When the actual copy number *N*_*G*_ is 13, the possibilities of getting a certain *n*_*G*_ with different section thickness (*t*) and nuclear diameter (*d*) were described by Eqs (8–10), and illustrated in a series of curves (Fig. 3a–f). The black dashed vertical line is the *n*_*G*_ derived experimentally at thickness *t* = 4, diameter *d* = 3.35, which falls near the curve of the theoretical *t* = 4 and *d* = 3, indicating that the model is a reasonable approximation.

The model showed that the observed gene copy number *n*_*G*_ is influenced by three factors.

(i) Section thickness (*t*). The probability curve is shifted leftward with thinner sections (Fig. 3a–f), indicating more partial nuclei are present and therefore a more severe tendency to under-estimate the gene copy number.
(ii) Nuclear diameter (*d*). By comparing upper and lower panels, it is evident that for the same thickness, samples with smaller nuclei have larger observed copy number, as more complete nuclei are preserved on the slide.
(iii) Enumeration threshold (*c*). By changing *c* value from 0.7 to 0.9, that is, using stricter standards to omit incomplete nuclei, the probability peaks shifted toward a larger observed value.

A more straightforward illustration was shown as bubble graph in Fig. 3g, with the bubble size representing the probability of having an observed copy number *n*_*G*_ on sections of different thickness based on binomial distribution. The experimental data was shown as empty black circles, with their diameters representing standard deviation of triplicate experiments. Overall, the model matched the experimental data within the range of experimental error, except for very thicker sections. It is likely that the parameter *c* ≥ 0.8 may not be optimal for thick sections, since more complete cells are present (Fig. 2d) and a larger *c* value would be a better match to human observers.

### Probability for false negative results when using thinner sections

**Figure 4:**
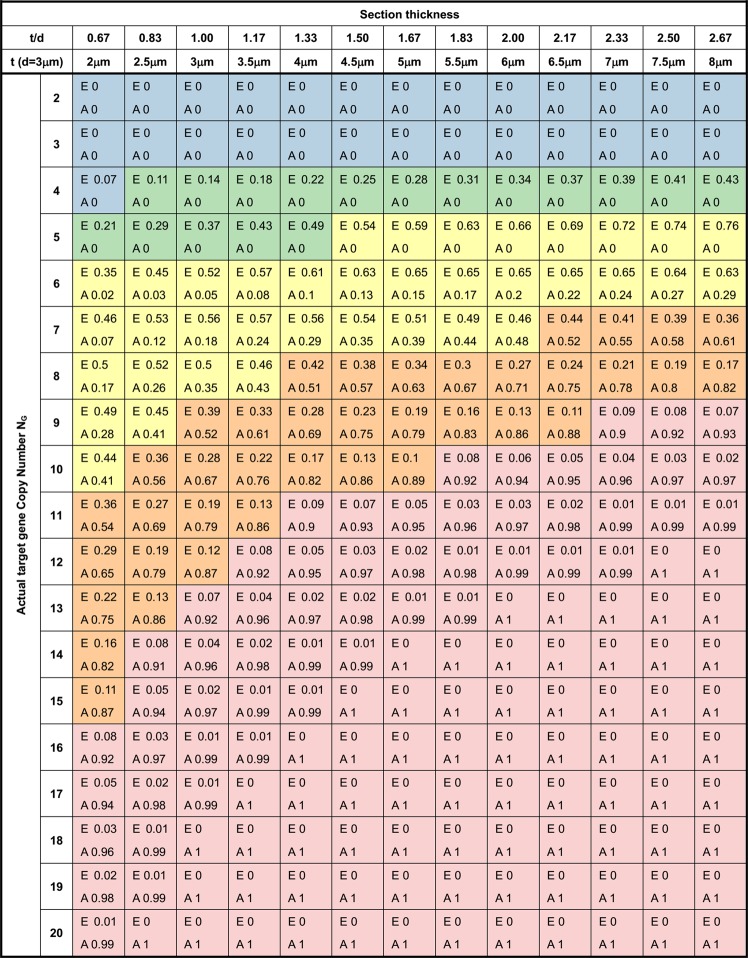
Probability of the observed results for sections of different thickness. The first column is the actual gene copy number of a specimen (*N*_*G*_). The first row is the section thickness that varies from 2 to 8 μm. Assuming the average nuclear diameter is 3 μm, and enumeration threshold *c* ≥ 0.8 (acceptable nucleus diameter is 80% of the full size, corresponding to apparent area of 64%), the probabilities of observing positive (*P*_*pos*_, *n*_*G*_ ≥ 6.0, shown as ‘A’ for amplification) or equivocal (*P*_*eqv*_, 4.0 ≤ *n*_*G*_ < 6.0, shown as ‘E’) results are shown. The different shadings are as following: Blue (*P*_*neg*_ ≥ 0.90); Green (0.5 ≤ *P*_*neg*_ < 0.9); Yellow (*P*_*neg*_ < 0.5 and *P*_*pos*_ < 0.5); Orange(0.5 ≤ *P*_*po*s_ < 0.9); Pink (*P*_*po*s_ ≥ 0.90).

Figure 4 showed the theoretical results for sections with different *t/d* (thickness to diameter) ratio at *c* ≥ 0.8. The probabilities for Equivocal (E) and Amplification (A) results are calculated for HER2. The model indicated that the chance for false negative results is increased when using thinner sections. For instance, if the nuclei diameter is 3 μm and the actual gene copy number *N*_*G*_ is 8, using 6 μm sections the probability of observing a positive result (*n*_*G*_ ≥ 6) is 71%, and for equivocal (4 ≤ *n*_*G*_ < 6) is 23%. When 2 μm sections are used, the probability for positive result is reduced to 17%, and for equivocal and negative increased to 50% and 33%, respectively, indicating that one would get wrong results by a larger chance.

### Correction for the results obtained using thinner sections

**Figure 5:**
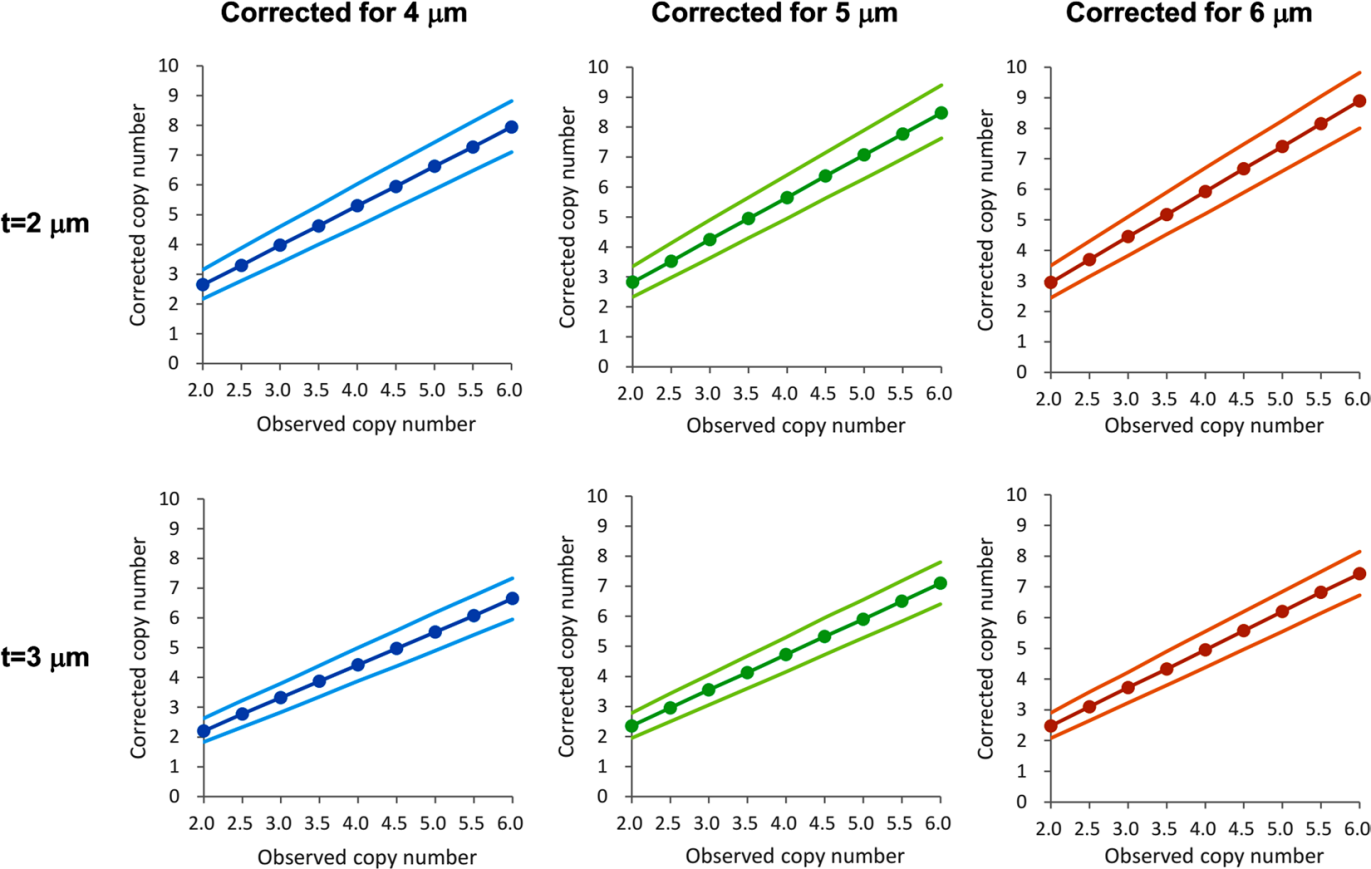
Copy number correction based on results from thinner sections. The corrected copy number for 4, 5, and 6 μm sections based on results from 2 or 3 μm sections were plotted, with the peak values of the probability curves shown as the filled circles. The faint lines above and below the peak curve represent 95% confidence intervals according to Table 4.

If the section thickness of the FISH/ISH experiment is known, the mathematical model can be used to correct for the results obtained from the improperly thin sections. Table 4 showed the corrected copy number for 4–6 μm sections given the results from 2 or 3 μm sections (or *t/d* ratio of 2/3 and 1, respectively) at *c* ≥ 0.8, assuming nuclear diameter *d* is 3 μm. The relationship for corrected copy number and observed copy number on thin sections were plotted in Fig. 5, with the 95% confidence interval shown above and below with fainter colors.

**Table 4:** Corrected copy number for 4–6 μm sections when observed copy number is obtained using 2 or 3 μm sections.

Observed	Corrected		
t = 2 μm	4 μm	5 μm	6 μm
2.0	2.65 (2.17–3.15)	2.83 (2.33–3.35)	2.95 (2.45–3.50)
2.5	3.30 (2.78–3.88)	3.53 (2.98–4.13)	3.70 (3.15–4.30)
3.0	3.98 (3.38–4.60)	4.25 (3.63–4.90)	4.45 (3.83–5.10)
3.5	4.63 (4.00–5.30)	4.95 (4.30–5.65)	5.18 (4.53–5.90)
4.0	5.30 (4.60–6.03)	5.65 (4.95–6.40)	5.93 (5.20–6.70)
4.5	5.95 (5.23–6.73)	6.38 (5.63–7.15)	6.68 (5.90–7.48)
5.0	6.63 (5.85–7.43)	7.08 (6.28–7.90)	7.40 (6.60–8.25)
5.5	7.28 (6.48–8.13)	7.78 (6.95–8.65)	8.15 (7.30–9.05)
6.0	7.95 (7.10–8.82)	8.48 (7.63–9.40)	8.90 (8.00–9.82)
**t** = **3 μm**	**4 μm**	**5 μm**	**6 μm**
2.0	2.20 (1.83–2.63)	2.35 (1.95–2.78)	2.48 (2.08–2.90)
2.5	2.78 (2.33–3.23)	2.95 (2.50–3.43)	3.10 (2.65–3.58)
3.0	3.33 (2.83–3.80)	3.55 (3.05–4.05)	3.73 (3.23–4.22)
3.5	3.88 (3.35–4.40)	4.13 (3.60–4.68)	4.33 (3.80–4.90)
4.0	4.43 (3.88–5.00)	4.73 (4.15–5.30)	4.95 (4.38–5.55)
4.5	4.98 (4.38–5.58)	5.33 (4.72–5.95)	5.58 (4.97–6.20)
5.0	5.53 (4.90–6.18)	5.90 (5.28–6.55)	6.20 (5.55–6.85)
5.5	6.08 (5.43–6.75)	6.50 (5.83–7.18)	6.83 (6.15–7.50)
6.0	6.65 (5.95–7.33)	7.10 (6.40–7.80)	7.43 (6.73–8.15)

The first column is the observed gene copy number using 2 μm or 3 μm sections, respectively. The corrected copy number were shown for 4, 5 and 6 μm sections, with the 95% confidence interval in parentheses. The calculation is based on enumeration threshold c ≥ 0.8 and nuclear diameter of 3 μm.

From Table 4 and Fig. 5, it is evident that, for instance, when the HER2 copy number is 4.5 (equivocal) on 2 μm section, the corrected results on 4 to 6 μm sections are largely positive. For MET, the observed copy number of 4.0 on 2 μm section, which is apparently a negative result, corresponds to a positive result (>5.0) on 4–6 μm sections. Table 4 and Fig. 5 apply generally for correcting the influence of section thickness on gene copy number measurements by FISH/ISH.

## Discussion

In this study, we showed that section thickness of FFPE slides influences FISH assay for gene copy number measurement and consequently gene amplification status. This conclusion is different from that of Babic and colleagues^21^, which concluded that changing section thickness from 2 to 8 μm did not affect FISH results. The authors did notice, however, a trend of reduced HER2 and CEP17 copy number on thinner sections for the majority of the specimens. Especially for the human case 2^21^, the observed HER2 copy numbers were 3.0, 4.0, 4.3, and 5.1 on sections of 2 to 8 μm, respectively. It would have been judged as negative or equivocal based on ASCO/CAP guideline^2–4^, which is consistent with the current study. The authors concluded, however, that the difference did not reach statistical significance and therefore the section thickness did not affect FISH results. Theoretically, the influence of section thickness on FISH result cannot be ruled out, since the statistics for the reciprocal conclusion was not reported.

In the current study, we also found that the observed gene copy number is positively correlated with section thickness. Due to errors of the measurement, not all data reached statistical significance. Hence, a mathematical approach was adopted to reveal the nature of the influence of section thickness on FISH. The mathematic model showed that the presence of partial nuclei is the underlying cause for underestimating gene copy number, which subsequently leads to increased chance for false negative results when using thinner sections.

It would be optimal if the FFPE section thickness is measured as a pre-analytical control step. There is no easy way, however, to do so. Hand-held industrial thickness gauge cannot reach sub-micron precision. Using microscope to measure thickness based on focal planes with the fine focus dial is prone to mechanical error. We also considered whether choosing a larger enumeration threshold *c* would be beneficial. Figure 2d showed that even if *c* = 1, which means only “complete” nuclear images are counted, not all of them have a full volume. Hence, counting large nuclei will alleviate but not solve the problem of underestimating the gene copy number on thinner sections.

Notably, although the observed gene copy number is influenced by section thickness, the ratio between the target and control signals is unaffected. Based on the mathematic model, the partial nuclei would affect both target and control signals proportionally. Nevertheless, partial nuclei would result in fewer signals per image, which based on Poisson distribution would result in larger random variations. In this sense, thicker sections with more complete nuclei are still recommended.

Moreover, the measurement gene copy number *per se* is important, as it is also used as an independent factor for prognosis and selection for targeted therapy, for HER2^2–4^, MET^6–8,16,22^, and EGFR^11,12^. When using thinner sections, some patients may be denied of the opportunity for targeted therapy, for instance, trastuzumab for HER2 positive breast cancer and gastric cancer, and crizotinib for MET positive lung cancer. Therefore, the danger of using thinner sections should be emphasized to ensure accurate lab practice and quality patient care.

As thin sections of 2–3 μm is widely used for HE and IHC, if only these sections are available in clinical laboratory for FISH/ISH, the mathematic model can be used to make corrections (Table 4 and Fig. 5). The correction can also be used to re-analyze historical data from thinner sections. One should, however, proceed with caution. The model makes several assumptions, such as regarding the nuclei as spheres with a uniform diameter, and the distribution of signal dots are random. In reality, the nuclei are often ellipsoid with a range of diameters. The genome is organized nonrandomly within the nucleus, with heterochromatin usually localized to the nuclear periphery^23,24^. With these limitations in mind, one can use the correction table (Table 4 and Fig. 5) to estimate the extent of deviation in general, for genes such as HER2, MET, EGFR, FGFR, and MYC etc., and to persuade laboratories to adhere to the guideline thickness.

Would using 4 vs. 6 μm sections make a difference? For highly amplified genes whose actual copy number is >10, the chance for false negative is low for both 4 and 6 μm sections (Fig. 4). For those with actual copy number between 6 and 10, however, the probability of false negative or equivocal results are much higher for 4 μm sections. Hence 6 μm is preferred. When the sections are too thick, however, the nuclei may overlap, and dewaxing may be incomplete^21^. In general, using thicker sections and avoiding enumerating the partial nuclei would maximize the chance to obtain a good approximation to the true value.

For research, assay development and clinical trials that utilizes FISH/ISH assay for gene copy number measurement, the section thickness should be regarded as an important parameter to control, especially for multi-center studies. As the microtomes are not calibrated for section thickness, the sections obtained according to a specified interval, for instance, 4~6 μm, may have even wider actual range of thickness. Hence, it is suggested that during research and clinical trials, especially the costly multiple centers studies, a fixed thickness should be specified to reduce experimental variation and increase statistical power to obtain a definitive outcome.

## Methods

### Model for FISH signals distribution in sections of different thickness

FISH Signals Distribution Model was programmed with Matlab R2016a software.

Cell nuclei are modeled as spheres with an average diameter *d*. The volume of a complete nucleus (*V*_*0*_) is:

1 $$C{V}_{0}=\frac{1}{6}\pi {d}^{3}$$

When a FFPE block is sectioned to thickness *t*, the partial nuclei on the slide is illustrated as shaded regions in Fig. 2a. The depth (*x*) of a partial nucleus is defined as the distance from the upper surface of paraffin to the bottom of the nucleus. The volume of the partial nucleus *V*_*x*_ is:

2 $${V}_{x}=\pi (\frac{d}{2}{x}^{2}-\frac{1}{3}{x}^{3})$$

To calculate the volume of partial nucleus remained on the slide, two scenarios are considered.

If *t* ≥ *d* (Fig. 2b, section thickness is greater than nuclear diameter), the nuclear volume remained on the slide is:

3 $$V(t\ge d)=\{\begin{array}{c}\pi (\frac{d}{2}{x}^{2}-\frac{1}{3}{x}^{3}),\,\,0\le x\le d\\ \frac{1}{6}\pi {d}^{3},\,d < x\le t\\ \frac{1}{6}\pi {d}^{3}-\pi [\frac{d}{2}{(x-t)}^{2}-\frac{1}{3}{(x-t)}^{3}],\,t < x\le t+d\end{array}$$

If *t* < *d* (Fig. 2c), the nuclear volume remained on the slide is:

4 $$V(t < d)=\{\begin{array}{c}\pi (\frac{d}{2}{x}^{2}-\frac{1}{3}{x}^{3}),\,\,0\le x\le t\\ \pi (\frac{d}{2}{x}^{2}-\frac{1}{3}{x}^{3})-\pi [\frac{d}{2}{(x-t)}^{2}-\frac{1}{3}{(x-t)}^{3}],\,t < x\le d\\ \frac{1}{6}\pi {d}^{3}-\pi [\frac{d}{2}{(x-t)}^{2}-\frac{1}{3}{(x-t)}^{3}],\,d < x\le t+d\end{array}\,$$

As the human observer would not count the very small nuclei, in the model signals are enumerated only when nuclear image has a diameter greater than *d*_*e*_ (expected diameter), which is a certain fraction (*c)* of full diameter *d:*

5 $${d}_{e}=c\times d$$

Assuming nuclei distribute randomly on the vertical axis, the depth *x* has a range of:

6 $$x\in [\frac{d}{2}(1-\sqrt{1-{c}^{2}}),\,t+\frac{d}{2}(1+\sqrt{1-{c}^{2}})]$$

Let ${x}_{1}=\frac{d}{2}(1-\sqrt{1-{c}^{2}})$ and ${x}_{2}=t+\frac{d}{2}(1+\sqrt{1-{c}^{2}})$, then the average volume of nuclei in the paraffin section is:

7 $$\bar{V}=\{\begin{array}{c}\frac{1}{{x}_{2}-{x}_{1}}{\int }_{{x}_{1}}^{{x}_{2}}V(t\ge d)dx,\,t\ge d\\ \frac{1}{{x}_{2}-{x}_{1}}{\int }_{{x}_{1}}^{{x}_{2}}V(t < d)dx,\,t < d\end{array}$$

Assuming the FISH signals have a uniform distribution in nucleus, the average probability (*p*) of a signal appearing in a nucleus is proportional to nuclear volume:

8 $$p=\frac{\bar{V}}{{V}_{0}}$$

Let the actual gene copy number be *N*, and the observed copy number be *n*. The possibility (*P*) of observing *n*_*G*_ target gene signals or *n*_*C*_ control signals is:

9 $$P({n}_{G})={C}_{{N}_{G}}^{{n}_{G}}{p}^{{n}_{G}}{(1-p)}^{{N}_{G}-{n}_{G}}$$

10 $$P({n}_{C})={C}_{{N}_{C}}^{{n}_{C}}{p}^{{n}_{C}}{(1-p)}^{{N}_{C}-{n}_{C}}$$

### Cell culture

SK-BR-3 (CBP60413, Cobioer) and HEK293T (ATCC) cells were used as positive and negative control cell lines, respectively. SK-BR-3 cells were grown in McCoy’s 5A Medium with L-Glutamine (GIBCO), 10% FBS (GIBCO), 100 μg/ml penicillin and 100 μg/ml streptomycin (GIBCO). HEK293T cells were grown in DMEM with 4.5 g/L glucose, L-glutamine and sodium pyruvate (WISENT BIOPRODUCTS), 10% FBS (GIBCO), 100 μg/ml penicillin and 100 μg/ml streptomycin (GIBCO). All cells were cultured in a humidified 37 °C cell culture incubator with 5% CO_2_ (BB150, Thermo) in 100 mm × 20 mm cell culture dishes (43016710, Corning).

The cells were trypsinized, washed twice in PBS, and counted with hemacytometer (1492, Reichert Bright-Line) three times, and the cell concentrations were averaged if the relative standard deviation of the triplicate results was acceptable (CV < 10%).

### Control FFPE slide preparation

The cell pellets were fixed in 20 volumes of methanol (A601617, BBI) for 10 min at room temperature, washed with PBS, mixed with 4% melted agarose (75510-019, Invitrogen) and placed in a mold. After cooling for 5 minutes, agarose blocks were placed in plastic cassettes and underwent standard formalin fixation, dehydration and paraffin block preparation.

The paraffin blocks containing control cell lines were sections with HM325 rotary microtome (902100, Thermo) to obtain triplicates sections with different thickness.

### Cytogenetic slide preparation

The cells were resuspended in 10 mL 75 mM KCl and incubated in 37 °C water bath for 20 minutes. After addition of 1 mL Carnoy’s fixative, the tubes were mixed and centrifuged at 500 g for 10 min, and the supernatant were discarded. The cell pellet was resuspended in 10 ml Carnoy’s fixative, kept at room temperature for 10 min, and centrifuged. The cell pellet was washed again in Carnoy’s fixative, resuspended in 100 μl Carnoy’s fixative, and dropped onto ice-cold positively charged slides. The slides were air dried at room temperature before FISH experiment.

### FISH experiment

Vysis PathVysion HER-2 DNA Probe Kit II (06N46-030, Abbott) and Vysis Paraffin Pretreatment Reagent Kit II (07J02-002, Abbott) was used for HER2 FISH testing on breast cancer specimens. Vysis MET SpectrumRed FISH Probe Kit (06N05-020, Abbott) and Vysis Paraffin Pretreatment IV & Post-Hybridization Wash Buffer Kit (01N31-005, Abbott) were used for MET FISH testing on lung cancer specimens. FFPE slides were baked at 60 °C for 2 h, deparaffinized three times with xylene (10023418, Sinopharm Chemical Reagent CO., Ltd) for 10 min each, and immersed twice in 100% EtOH (80176961, Sinopharm Chemical Reagent CO., Ltd) for 1 min each. After air dry. The slides were incubation in pre-treatment solution at 80 °C for 20 min, followed by protease digestion for 30 min at 37 °C. Slides were dehydrated in a graded ethanol series of 70%, 85% and 100% and air dried.

Probe mixture was added onto the hybridization area, then cover-slipped and sealed with rubber cement. Slides were incubated in Thermobrite (Abbott) at 73 °C for 5 min (HER2) or 3 min (MET) for denaturation, and hybridized at 37 °C overnight. After gently removing the rubber cement and cover slip, the slides were washed in Washing Buffer II (HER2) at 72 °C for 2 min or Washing Buffer II (MET) at 74 °C for 2 min. Then the slides were washed briefly in 70% EtOH, air dried in darkness, and stained with DAPI counter stain and cover-slipped. FISH results were examined with BX43 fluorescence microscope (Olympus) and photographs were taken with digital camera (CellSens) by using appropriate filters.

### Measurement of nuclear diameter

Cytogenetic or FFPE slides were dewaxed, rehydrated in ethanol gradient, and stained with DAPI counter stain. The slides were examined with BX43 fluorescence microscope (Olympus) and photographs as mentioned above.

### Statistics

ANOVA test were performed for gene copy number measurement from sections of different thickness using Graphpad Prism 5.

## Data Availability

The Matlab program of the model is available upon request.
